# Mpox Pharyngitis

**DOI:** 10.1007/s12070-024-04567-1

**Published:** 2024-03-02

**Authors:** Miguel Saro-Buendía, Rodolfo David Palacios-Díaz, Pedro Suárez-Urquiza, Miguel Mansilla-Polo, Catalina Bancalari-Díaz, Javier Cabrera-Guijo, Miguel Armengot-Carceller

**Affiliations:** 1 Otorhinolaryngology- Head and Neck Surgery, La Fe University and Polytechnic Hospital, Valencia, Spain; 2 Dermatology, La Fe University and Polytechnic Hospital, Valencia, Spain; 3 Microbiology, La Fe University and Polytechnic Hospital, Valencia, Spain; 4 Departamento de Cirugia, Facultat de Medicina i Odontologia, Universitat de València (España), Valencia, Spain; 5 BMCG, Instituto de Investigación Sanitaria La Fe, CIBERES, Valencia, Spain; 6 Instituto de Investigación Sanitaria (IIS) La Fe, Valencia, Spain

**Keywords:** Mpox, Monkeypox virus, Mpox pharyngitis, Case report

## Abstract

A case of mpox pharyngitis in absence of cutaneous lesions is reported. Usually, clinical presentation is either a cutaneous eruption or a combination of cutaneous and mucosal lesions. In patients with atypical pharyngitis, regardless of the presence of skin lesions, pharyngeal swabs should be collected to rule out mpox.

## Introduction

Monkeypox (recently renamed as mpox) is caused by a zoonotic orthopox virus known as monkeypox [1]. It is endemic in central and occidental Africa. In 2022, it caused a global outbreak considered by the World Health Organization as a public health emergency of international concern [2]. Its transmission is mainly human-to-human by large respiratory droplets and direct contact with mucocutaneous lesions. Classically, it is presented with fever, lymphadenopathy, and a generalized cutaneous eruption. However, patients infected since the 2022 global outbreak showed more localised cutaneous lesions at different evolution stages and, less frequently, mucosal lesions [2–5]. In the absence of cutaneous lesions, diagnosis may be challenging. We share a case of mpox presented with acute pharyngitis, initially in absence of typical cutaneous lesions.

## Case Report

**Fig. 1:**
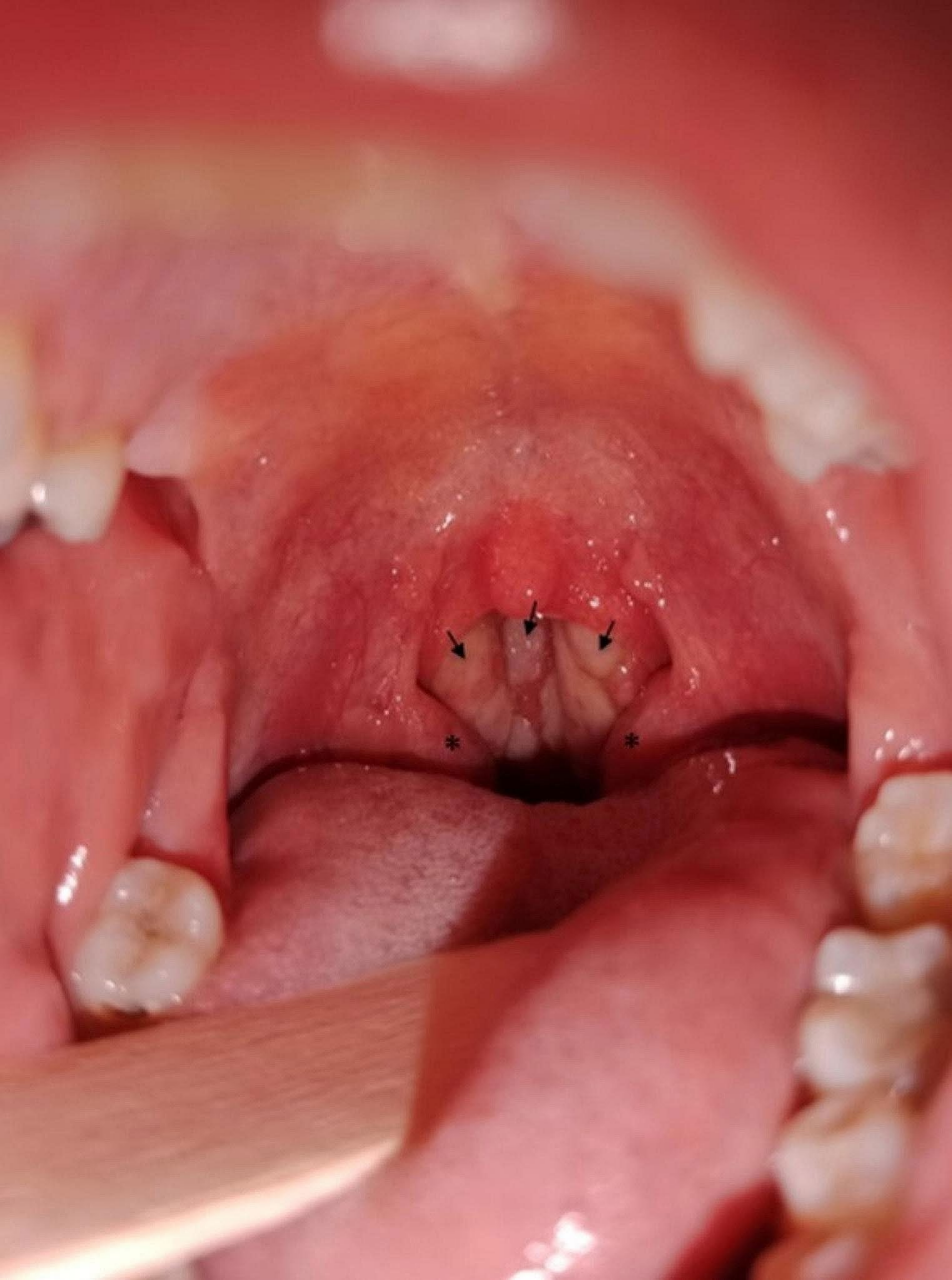
Examination of the oral cavity and oropharynx upon emergency department admission. Erythema and white ulcerative lesions in the posterior and lateral oropharyngeal walls (*arrows*). Tonsils do not seem affected (*)

**Fig. 2:**
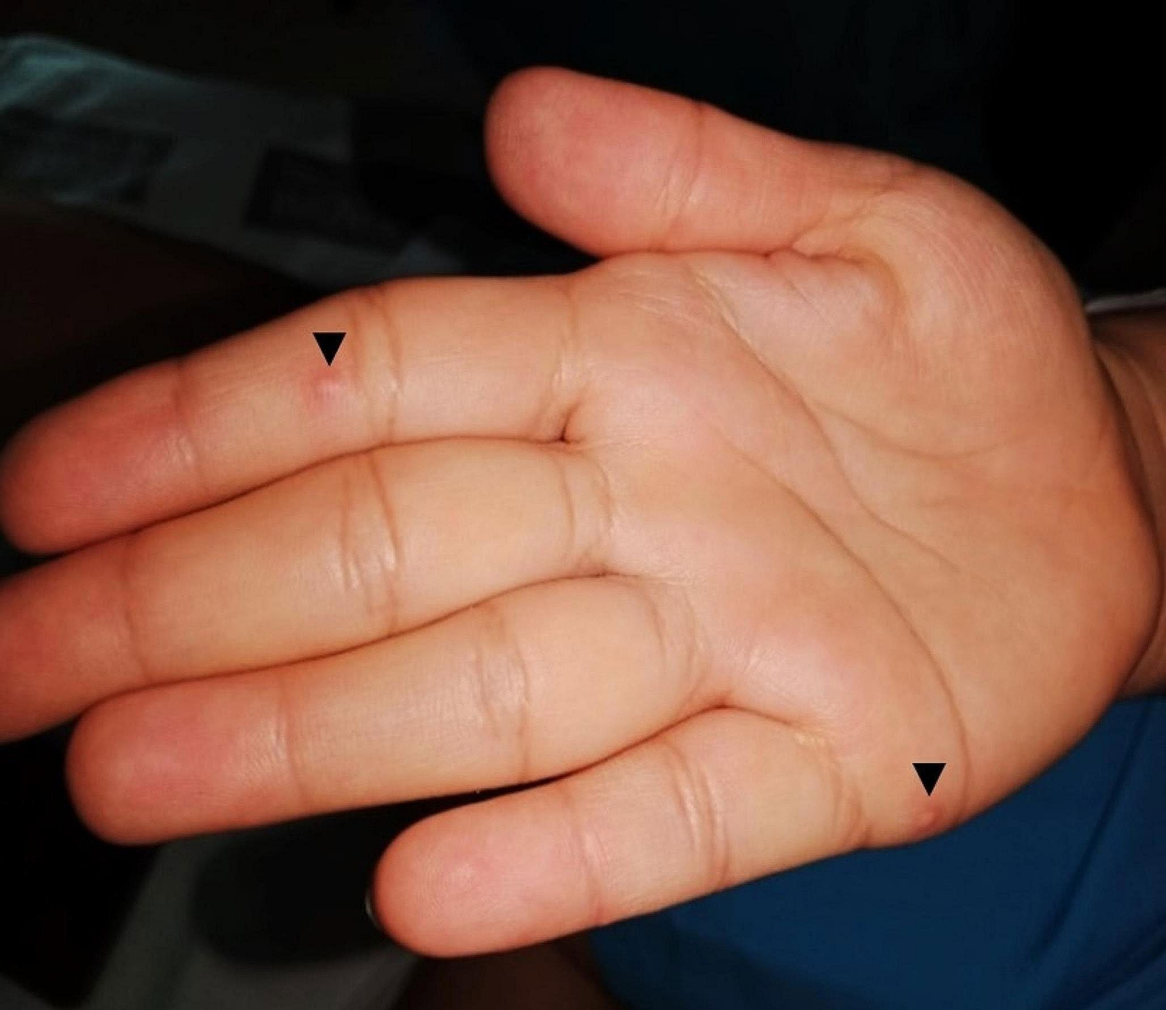
Vesicles on the palm of the right hand (*arrowheads*). These lesions appeared nineteen days after the onset of odynophagia

A 32-year-old female presented to our emergency department with odynophagia, bilateral neck pain and persistent fever for 15 days. She had not improved after a 7-day course of oral co-amoxiclav 875/125 mg every 8 h. On direct oropharyngeal examination, erythema and white ulcerative lesions in the posterior and lateral oropharyngeal walls were observed **(**Fig. 1**)**. From these lesions, swabs were collected. Endoscopy showed similar findings in the nasopharyngeal region, with intense swelling and erythema. Moreover, lesions in the posterior aspect of the left tonsil were observed. Cutaneous eruption or genital lesions were not observed. Computed tomography ruled out related complications and showed bilateral cervical lymphadenopathy. However, 4 days later (after 19 days with symptoms) the patient presented with pruritic vesicles and oedematous papules with umbilication and, in some of them, central crusting. These lesions were located under the lower lip, in both upper extremities (including hands), right scapula and right breast **(**Fig. 2**)**. Under suspicion of varicella infection, skin and mucosal swabs were collected and acyclovir 10 mg per kg every 8 h was started. However, three days later, polymerase chain reaction discarded varicella infection and, instead, monkeypox virus DNA was found. At confirmation, most lesions were resolving so specific antiviral therapy was not needed. A superadded bacterial infection was detected (*Streptococcus dysgalactiae* and *Staphylococcus aureus* grew from initial pharyngeal swabs) and treated combining intravenous co-amoxiclav and linezolid. Recommendations to avoid further spread of the virus were given by the department of preventive medicine and satisfactory clinical recovery was observed after two weeks.

## Discussion

Pharyngeal symptoms in the absence of cutaneous lesions are the initial clinical presentation of mpox infection in 4.9% of the patients, being odynophagia present in 16.8% [1, 5]. Tonsillitis occurs in 10.5% of the patients. The presence of white ulcerative lesions on the tonsillar surface has been described. These patients usually test negative for group A streptococcal antigen and report receptive oral sex, although our patient apparently did not [3]. Related pharyngeal complications like peritonsillar abscesses have been described [6]. Our patient presented white ulcerative lesions, mainly located in the lateral and posterior walls of nasopharynx and oropharynx. The distribution of the lesions differed from reported cases of mpox pharyngitis: tonsils were almost unaffected (except for a 2-millimeter ulcer in the posterior aspect of the left tonsil) and nasopharyngeal swelling was the main finding. Due to absence of specific features of mpox pharyngitis, this entity was not suspected until the onset of cutaneous lesions 19 days later. Similar to the majority of the cases, our case was not severe, and thus antiviral therapy with Tecovirimat or other agents was not required.

## Conclusions

These days, otolaryngologists and emergency physicians should suspect mpox in patients presenting atypical acute pharyngitis with white ulcerous lesions, even in the absence of cutaneous lesions. Antiviral therapy is rarely needed, but an early mpox diagnosis is essential to apply preventive medicine measures and control spreading of this disease.
